# Assessment of natural radioactivity in urban soil samples from Dhaka city and its associated health hazard

**DOI:** 10.1371/journal.pone.0345030

**Published:** 2026-03-19

**Authors:** Shikha Pervin, Nadia Sarker, Md. Masum Haider, Shanjib Karmaker, Tanzeem Tahmeed Reza, Selina Yeasmin, Mayeen Uddin Khandaker

**Affiliations:** 1 Applied Physics and Radiation Technologies Group, CCDCU, Faculty of Engineering and Technology, Sunway University, Bandar Sunway, Selangor, Malaysia; 2 Health Physics Division, Atomic Energy Centre, Bangladesh Atomic Energy Commission, Dhaka, Bangladesh; 3 Department of Physics, Mawlana Bhashani Science and Technology University, Santosh, Tangail, Bangladesh; 4 Nuclear Power and Energy Division, Bangladesh Atomic Energy Commission, Dhaka, Bangladesh; 5 Department of Electronics and Telecommunication Engineering, Chittagong University of Engineering and Technology (CUET), Chittagong, Bangladesh; 6 Department of Physics, College of Science, Korea University, Seongbuk-gu, Seoul Republic of Korea; 7 Faculty of Graduate Studies, Daffodil International University, Savar, Dhaka, Bangladesh; Cameroon National Radiation Protection Agency, CAMEROON

## Abstract

Humans are constantly exposed to radiation from their natural environment including soil and gamma radiation has harmful effects on them, so determination of radioactivity concentration in soil are very important. The present study aims to measure the activity concentrations of naturally occurring radionuclides ^226^Ra, ^232^Th, and ^40^K in urban soil samples collected from thirty different areas of Dhaka city. The analyzed was performed using a High-Purity Germanium (HPGe) gamma-ray spectrometer. The results showed that the mean activity concentrations of ^226^Ra, ^232^Th, and ^40^K were found 24.2 ± 1.0 Bqkg^-1^, 52.0 ± 2.0 Bqkg^-1^, and 352 ± 11 Bqkg^-1^, respectively. The average concentrations of ^226^Ra and ^40^K fall below the internationally recommended safety limits of 35 Bqkg^-1^ and 400 Bqkg^-1^, respectively, while ^232^Th value exceeded the recommended limit of 30 Bqkg^-1^ by approximately 1.7 times. The mean value of radium equivalent activity (Ra_eq_) was calculated as 125.7 Bqkg^-1^, which was far below the global safety threshold of 370 Bqkg^-1^. The estimated outdoor effective dose rates were 0.070 mSvy^-1^ and below the worldwide recommended limit of 1 mSvy^-1^. Additionally, excess life time cancer risk (ELCR) was below than the internationally accepted limit of 0.29 × 10^−3^. All things considered, the study is the first comprehensive dataset of urban soil in the area and revealed that there are no immediate health dangers due to the low radioactive hazard indices.

## 1. Introduction

Determining the concentrations of radionuclides in soil, both man-made and natural is essential for establishing baseline levels, which are critical for tracking changes over time, especially in the event of radioactive releases. This information is vital for both environmental monitoring and public health protection [1,2]. In 2011, the accident at the Fukushima Daiichi Nuclear Power Plant highlighted transboundary nature of nuclear accidents by releasing massive amounts of radioactive material into the surrounding environment. Approximately 80% of this material was eventually transported into the Pacific Ocean, contaminating terrestrial and other ecosystems [3–5]. Because radionuclides are found in a variety of geological elements, including rocks, soil, sand, water, and coal, plants, animals, and even human tissues [6–11]. As a result, both the environment and human beings are continuously exposed to radiation [6,12]. Understanding ionizing radiation is crucial for human radiation exposure, in contrast to nonionizing radiation. Electromagnetic radiation emitted by radioactive materials or occasionally by nuclear events is known as ionizing radiation [7,13]. Soil, in particular, is a major source of natural radioactivity and plays a key role in the migration and transfer of radionuclides into the broader environment [14,15]. The natural radioactivity in soil, primarily due to the decay of radionuclides from the uranium and thorium series, is widely recognized as a fundamental indicator of radiological contamination [16]. Because radionuclides from rocks can enter soil through rain and water flow and interact with soil particles, soil has a significant impact on natural background radiation. Soil radioactivity is a persistent source of radiation and radionuclide mobility in the environment due to human activities including the disposal of industrial waste and the heavy use of phosphate fertilizers [17,18]. The region has a high background radiation level, or the amount of background radiation brought on by human activity, as well as possible exposure impacts for the general public and geological and environmental factors related to soil radioactivity [19,20]. People receive maximum external gamma radiation from soil due to naturally occurring radionuclides such as ^238^U, ^232^Th and ^40^K [6,21]. Soil can also absorb and retain artificial radionuclides such as ^137^Cs, which is often unevenly distributed across different soil depths and terrestrial and artificial radionuclides can enter the human body through direct contact or through the food chain [22,23]. Long-term exposure to uranium and thorium, especially by inhalation, can result in a number of health issues, including oral tissue necrosis, chronic lung conditions, acute leukopenia, and anemia [12]. While thorium exposure has been connected to leukemia and malignancies of the bone, kidney, liver, lung, and pancreas, radium inhalation or ingestion can cause tumors in the bones, skull, and nasal tissues [18]. In recent times, there have been no detailed investigations on urban soils in Dhaka city and their associated health hazard assessments. Even though Dhaka city is growing quickly, there is still no complete study of the natural radionuclides and the radiological risks they pose in its urban soils. To fill this gap, the current study conducts a comprehensive investigation utilizing numerous soil samples, high-resolution gamma spectrometry measurements, a thorough radiological risk assessment, and rigorous statistical analyses. This method helps us better understand activity concentrations in a city that is both very crowded and hasn’t been studied much in terms of radiation. Therefore, the objectives of this study are to quantify the specific activities of the naturally occurring radionuclides ^226^Ra, ^232^Th, and ^40^K in Dhaka soils and calculate the associated radiological hazard indices. This study can contribute to urban planning, environmental and public safety policy making.

## 2. Materials and methods

### 2.1. Study area

**Fig 1:**
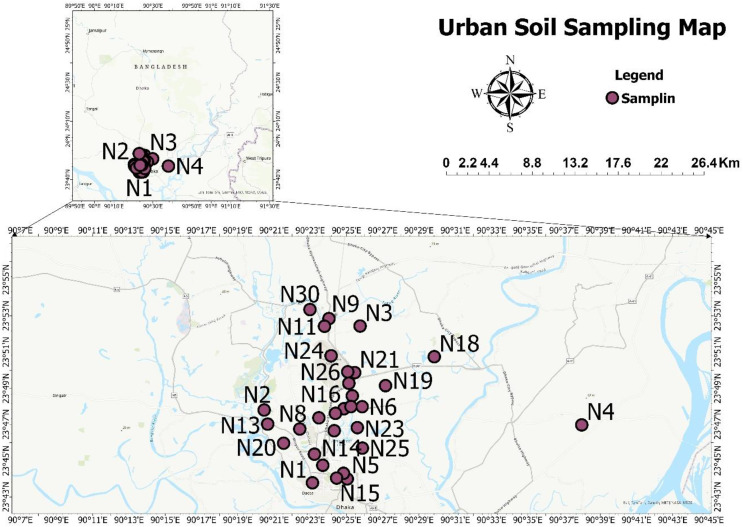
Location map of urban soil samples in Dhaka, Bangladesh.

Urban soil samples were gathered from thirty various areas in Bangladesh’s metropolis, Dhaka, for the study, and this city is the central zone of the country. The population of this city is approximately 1.25 million, according to a 2011 national survey [24]. This region was chosen because of its dense population, rapid urbanization, and risk of radioactive exposure from both natural and man-made sources. There are some nuclear medicine centers located in this city. Locations were chosen based on the accessibility of soil samples and to ensure a representative assessment of the area. Since the composition of soil and consequently, the concentration of radionuclides varies from one site to another, selecting multiple locations allowed for a more comprehensive understanding of how these variations influence radiological parameters such as effective dose rates and hazard indices. A location map of urban soil samples in Dhaka, Bangladesh, was created using ArcGIS Pro 2024 software and is shown in Fig 1.

### 2.2. Sample collection and processing

From the preceding study area, 30 urban soil samples were collected from 30 different locations of Dhaka city for instances near hospitals, university campus, school, commercial area, park, residence area, etc. Because the study aims to assess the general urban background rather than point out potential contamination sources. No formal permits or approvals were required prior to sample collection because all soil samples were collected from publicly accessible locations. The sampling was conducted exclusively in open public areas, and no samples were taken from private property, restricted zones, or protected sites. Therefore, approval from any regulatory or local authority was not necessary for this study. Each of the locations was considered to prepare one representative soil sample. During sample soil sample collection from Dhaka city, a stratified sampling approach was employed [25]. The soil samples were taken between 0 and 5 cm below the surface. In order to improve reproducibility and dependability, three replicate samples were taken from each site throughout the winter months of December 2023 to February 2024. Nearly 1 kg soil samples were collected in polyethylene bags, then properly marked and identified by their locations using the Global Positioning System (GPS). Then all collected samples were transferred to the environmental radiation & radioactivity laboratory of the Atomic Energy Centre, Dhaka.

During sample preparation, International Atomic Energy Agency (IAEA) guidelines were following [26]. All irrelevant components of the soil samples were removed, such as portions of stone, pebbles, leaves and roots, and then the mass of the sample was assessed after it had dried for a few days at room temperature and then oven-dried and achieved a steady mass. The samples were then ground into a fine powder and homogenized using a 2 mm sieve. For further examination, around 500 g of soil sample was transferred to a cylindrical pot. All the sample beakers were sealed tightly, wrapped with thick vinyl tape around the screw necks and stored for around 40 days to attain secular equilibrium between parents and their progenies [27,28].

### 2.3. Gamma ray spectrometry system

The activity concentrations of ^226^Ra, ^232^Th, and ^40^K in the soil samples were determined using a p-type High-Purity Germanium (HPGe) detector. The model of the detector was NATS GCD-30185, Baltic Scientific Instruments, Riga, Latvia. The diameter of the detector was 59.4 mm and the thickness was 56.6 mm. The operating bias voltage of the detector was + 2700, with a relative efficiency of 30% and an energy resolution of 1.67 keV full width at half maximum (FWHM) at the 1332 keV peak of ^60^Co. A 16k multi-channel analyzer and associated electronics for data acquisition of photo-peak areas were connected to the system. Gamma-ray spectra were analyzed using Spectra Line GP© software. Energy calibration was performed using gamma-ray point source (^137^Cs and ^60^Co) and efficiency calibration was performed by using a standard reference material which code was 8501-EG-SVE, Eckert & Ziegler Analytics along with a multi-nuclide gamma-ray source ^252^Eu [29].

A background count was also taken using an empty container under identical conditions. The counting time for both background and sample were 20000 seconds. The counts of the background spectrum were subtracted from the counts of the sample spectrum to obtain final counts, from which the activities of each radionuclide were calculated.

For spectral analysis, the single gamma-ray line at 1460.822 keV was used to determine the activity concentration of ^40^K. The activity concentration of ^226^Ra was assessed using the photo-peaks at 295.221 keV and 351.922 keV from ^214^Pb, and 609.320 keV, 1120.310 keV, and 1764.551 keV from ^214^Bi. The activity of ^232^Th was derived from the photo-peaks at 238.630 keV and 300.087 keV from ^221^Pb, 911.205 keV and 968.970 keV from ^228^Ac, and 583.190 keV and 2614.533 keV from ^208^Tl [30]. However final activity was calculated using weighted mean approach considering all gamma lines [3]. From sample collection to counting precautions were taken to prevent any form of contamination.

### 2.4. Calculation of activity

The minimum detectable activity (MDA) represents the lowest activity level that the detection system can reliably measure. Any sample with a count rate below this threshold may yield a negative value, which is not accurate. The MDA for each radionuclide in the detector system can be calculated using the following formula [31,32]:


$$\text{MDA}=\frac{\text{4}.\text{653 }\times \;\sigma_{B}\;+\;\text{2}.\text{706}}{t\;\times \;\in (E)\;\times \;P_{\gamma }(E)\;\times \;W}\;$$ (1)

Where, 𝜎𝐵 denotes the standard deviation of the background count rate obtained from measurements conducted under identical conditions as the samples, but without any radioactive source or sample present, t is the counting time, ∈(E) is absolute efficiency at photon energy E, $P_{\gamma }(E)$ is the gamma photon emission probability at the gamma line corresponding to peak energy and w is the mass of the soil sample. For ^226^Ra, ^232^Th and ^40^K at 95% confidence for detector, the MDA was 0.33, 0.08 and 0.38 respectively for soil samples. By subtracting the required peak energy area from a linear background distribution of pulse spectra, the net sample amount was computed. It was possible to derive activity levels for each sample based on their net counts using the following formula [33]:


$$S=\frac{CPS}{\varepsilon \times P_{\gamma }\times m(kg)}$$ (2)

here, S represents the activity concentration of the soil sample in Bqkg^-1^, CPS is the count rate (count per seconds), ε represents the efficiency of the gamma energy, $P_{\gamma \;}$ gives the absolute intensity of γ ray, and m represents the sample mass in kilograms.

The uncertainty associated to the activity concentration of a radionuclide (ΔS) can be shown by following formula [3,34]:


$$\frac{\Delta S}{S}\;=\;\sqrt{{\left(\frac{\Delta N}{N}\right)}^{\text{2}}+\;{\left(\frac{\Delta P_{\gamma }}{P_{\gamma }}\right)}^{\text{2}}+\;{\left(\frac{\Delta \in }{\in }\right)}^{\text{2}}+{\left(\frac{\Delta M}{M}\right)}^{\text{2}}+\;{\left(\frac{\Delta t}{t}\right)}^{\text{2}}}$$ (3)

Here, $\frac{\Delta N}{N}$ is the counting statistical uncertainty, $\frac{\Delta P_{\gamma }}{P_{\gamma }}$ is the uncertainty in gamma emission probability, $\;\frac{\Delta \in }{\in }$ is the uncertainty in detector efficiency and $\;\frac{\Delta M}{M}\;$ is the uncertainty in sample mass and $\frac{\Delta t}{t}$ is the uncertainty in time.

Next, weighted mean method was applied to obtain the final concentration of activity ($S_{m}$± $\sigma S_{m}$) By integrating the different activity concentrations (S ± σS_m_) obtained from each distinct gamma-line. The weighted mean activity (Sm ± σSm) was calculated using the following formula [3]:


$$S_{m}=\;\frac{\sum_{i}\left(W_{i}\times S_{i}\right)}{\sum_{i}W_{i}}$$ (4)

where $S_{m}$ is the weighted mean activity, and $S_{i}$ is the measured activity concentration for the i-the gamma line. $W_{i}$ is the weighted factor.

The weighted mean’s standard error is provided by [3]:


$$\sigma S_{m}=\sqrt{\frac{\text{1}}{\sum_{i}W_{i}}}$$ (5)

where σi is the appropriate uncertainty for the gamma line.

### 2.5. Radium equivalent activity (Ra_eq_)

The radium equivalent activity is a widely used index for comparing the specific activities of materials containing ^226^Ra, ^232^Th and ^40^K. It is calculated using the following expression as [35]:


$${Ra}_{eq}=S_{Ra}+{\text{1}.\text{4}\text{3}S}_{Th}+{\text{0}.\text{0}\text{7}\text{7}S}_{K}$$ (6)

Where, Ra_eq_ is the radium equivalent activity in Bqkg^-1^. S_Ra_, S_Th_ and S_K_ are the activity concentrations (in Bqkg^-1^) of ^226^Ra, ^232^Th and ^40^K, respectively.

### 2.6. Absorbed dose rate

The concentration of radionuclides in the soil determines how much the natural radionuclides contribute to the absorbed dose rate in the air. Based on the radionuclides in the soil, absorbed dose rate conversion factors can be used to calculate the dose. The following formula is used to get the outdoor gamma absorbed dose rate using the conversion variables specified in UNSCEAR 2000 [6]:


$$D_{out}={\text{0}.\text{4}\text{6}\text{2}S}_{Ra}+\text{0}.\text{6}\text{0}\text{4}S_{Th}+\text{0}.\text{0}\text{4}\text{1}\text{7}S_{K}$$ (7)

Where, $D_{out}\;$is the absorbed dose rate in air in nGyhr^-1^. S_Ra_, S_Th_ and S_K_ are the activity concentrations of ^226^Ra, ^232^Th and ^40^K in Bqkg^-1^.

### 2.7. Annual effective dose equivalent

Using the UNSCEAR (2000) conversion coefficient of 0.7 SvGy ⁻ ¹ and an outdoor occupancy factor of 0.2, the absorbed dose rate in air was converted to the annual effective dose equivalent (AEDE), assuming that people spend, on average, 20% of their time outdoors. Annual effective dose equivalent (AEDE) for outdoor was calculated using the following formula [6,12]:


$${AEDE}_{Out}=D_{out}\times \text{8}\text{7}\text{6}\text{0}\times \text{0}.\text{2}\times \text{0}.\text{7}\times {\text{1}\text{0}}^{-\text{6}}$$ (8)

where, ${AEDE}_{Out}$ is the annual effective dose equivalent (AEDE) for outdoor (mSvy^-1^), $D_{out}$ is the outdoor absorbed dose rate (nGyhr^-1^), 8760 is the number of hours in a year, 0.7 is the dose conversion coefficient for absorbed dose to effective dose (SvGy^-1^), 0.2 is the outdoor occupancy factor, 10^−6^ is the unit conversion factor from nGy to mSv.

### 2.9. Excess lifetime cancer risk

Examining the correlation between a populations lifetime cancer development likelihood and anticipated consumption of particular substances or exposure to particular circumstances can yield the excess lifetime cancer risk (ELCR) [36]:


$$ELCR=AEDE\times D_{L}\times R_{F}\times {\text{1}\text{0}}^{-\text{3}}$$ (9)

Where, AEDE is the annual effective dose rate equivalent (mSvy^-1^), D_L_ is the duration of life (70 years) [18] and R_F_ is the risk factor (0.05 Sv^-1^ recommended by ICRP) associated with radiation exposure [37] and ${\text{1}\text{0}}^{-\text{3}}$ is used for convert mSv to Sv.

## 3. Results and discussion

### 3.1 Activity concentration

Urban soil samples were measured by using gamma ray spectrometry system for radioactivity analysis. Table 1 demonstrates activity concentrations of ^226^Ra, ^232^Th and ^40^K in thirty soil samples collected from different locations of Dhaka city.

**Table 1:** Activity concentrations of ^226^Ra, ^232^Th and ^40^K from the collected urban soil of Dhaka city.

Sample ID	Location	Latitude	Longitude	Activity concentration (Bqkg^-1^)		
				^226^Ra	^232^Th	^40^K
N1	Azimpur	23.72980∘N	90.3854°E	20.9 ± 1.0	51.0 ± 3.0	360 ± 11
N2	Diabari	23.7967∘N	90.3410∘E	24.6 ± 1.0	49.0 ± 3.0	490 ± 12
N3	Uttarkhan	23.8740∘N	90.4292°E	27.0 ± 0.2	50.0 ± 3.0	280 ± 12
N4	Jatrabari	23.7830∘N	90.6330°E	23.0 ± 1.0	52.0 ± 2.0	280 ± 11
N5	Motijheel	23.73333°N	90.417458°E	25.0 ± 0.01	58.0 ± 3.0	460 ± 12
N6	Notun Bazar	23.799761°N	90.431138°E	21.5 ± 1.0	56.0 ± 3.0	310 ± 12
N7	Shantinagar	23.73854°N	90.41392°E	22.4 ± 1.0	46.0 ± 3.0	470 ± 12
N8	Agargaon	23.7792°N	90.3737∘E	35.0 ± 1.0	55.0 ± 3.0	530 ± 12
N9	Abdullahpur	23.88084°N	90.40059∘E	25.0 ± 1.0	56.0 ± 3.0	311 ± 11
N10	Bangla Motor	23.74584°N	90.39494∘E	22.5 ± 0.4	55.0 ± 3.0	293 ± 11
N11	Uttara	23.873751°N	90.396454∘E	19.4 ± 0.04	58.0 ± 3.0	440 ± 11
N12	Mohakhali	23.777628°N	90.405449∘E	24.0 ± 1.0	54.0 ± 3.0	320 ± 11
N13	Gabtoli	23.783726°N	90.344246∘E	30.0 ± 0.1	62.0 ± 2.0	360 ± 11
N14	Farmgate	23.7561067°N	90.387196∘E	18.6 ± 1.0	35.0 ± 2.0	340 ± 12
N15	Shegunbagicha	23.734032°N	90.4076745∘E	31.0 ± 1.0	57.0 ± 2.0	320 ± 12
N16	Gulshan	23.797911°N	90.414391∘E	13.8 ± 0.4	32.0 ± 2.0	318 ± 11
N17	Nadda	23.80982°N	90.42206∘E	34.0 ± 0.2	57.0 ± 2.0	340 ± 12
N18	Purbachal	23.845777°N	90.497436∘E	19.9 ± 1.0	53.0 ± 3.0	410 ± 12
N19	Bashundhara	23.81910°N	90.45260∘E	34.0 ± 0.3	63.0 ± 3.0	360 ± 12
N20	Mohammadpur	23.76620°N	90.35890∘E	19.7 ± 0.2	36.0 ± 3.0	298 ± 12
N21	Khilkhet	23.83110°N	90.42430∘E	19.3 ± 1.0	48.0 ± 2.0	260 ± 12
N22	Baridhara	23.79990°N	90.42080∘E	20.4 ± 0.3	34.0 ± 3.0	360 ± 11
N23	Badda	23.780546°N	90.426659∘E	27.0 ± 0.3	59.0 ± 3.0	330 ± 11
N24	Airport	23.846615°N	90.4026234∘E	24.4 ± 0.1	55.0 ± 2.0	303 ± 11
N25	Banasree	23.76171°N	90.43128∘E	25.0 ± 1.0	60.0 ± 3.0	310 ± 11
N26	Nikunja	23.8319°N	90.4178∘E	22.0 ± 1.0	54.0 ± 3.0	290 ± 11
N27	Kuril Bishwa Road	23.82136°N	90.41887∘E	27.0 ± 1.0	66.0 ± 2.0	350 ± 12
N28	Banani	23.7937°N	90.4066∘E	23.5 ± 1.0	55.0 ± 2.0	313 ± 12
N29	Kafrul	23.7896°N	90.3913∘E	21.2 ± 0.3	47.0 ± 3.0	510 ± 11
N30	Kamarpara	23.8892°N	90.3831∘E	25.0 ± 1.0	48.0 ± 3.0	253 ± 11
Average				24.2 ± 0.4	52.0 ± 3.0	352 ± 11
Skewness				+0.584	−0.997	1.057
Kurtosis				+0.397	+0.632	+0.156
Shapiro–Wilk test				0.950 (*p* > 0.22)	0.965 (*p* > 0.45)	0.940 (*p* > 0.15)

From Table 1, it has been seen that the activity concentration in soil samples varied in the range of 13.8 ± 0.4 (N16 samples from Gulshan) to 35.0 ± 1.0 Bqkg^-1^ (N8 sample from Agargaon) for ^226^Ra, 32.0 ± 2.0 (N16 from Gulshan) to 66.0 ± 2.0 Bqkg^-1^ (N27 from Kuril Bishwa Road) for ^232^Th, and 253 ± 11 (N30 sample from Kamarpara) to 530 ± 12 Bqkg^-1^ (N8 sample from Agargaon) for ^40^K respectively. The studied average ^226^Ra activity concentration found 24.2 ± 0.1 Bqkg^-1^ which was less than the global average of 35 Bqkg^-1^, the average activity concentration of ^232^Th estimated in this study was 52.0 ± 3.0 Bqkg^-1^ and thus much higher than the global average value of 30 Bqkg^-1^ and for ^40^K the average activity concentration estimated in this study was 352 ± 11 Bqkg^-1^ which was also below the global average value of 400 Bqkg^-1^. From Table 1 clearly demonstrates that the order of radionuclide abundance in Dhaka city urban soil was ^40^K > ^232^Th > ^226^Ra. Some locations (N2, N5, N7, N8, N11, N18, N29), the activity concentration of ^40^K exceeded the recommended global limit, likely due to abundance of ^40^K in rock and minerals and weathering, anthropogenic activities, soil composition [32]. Higher ^232^Th activity concentration in the soil sample caused by a number of things, including urbanization, anthropogenic activities, presence of thorium bearing mineral geology and erosion and weathering process [23,38]. Additionally, radium can move easily in environment and also the abundance of thorium is higher than uranium in earth crust [6].

Table 1 also shows that the skewness values of +0.584 for ^226^Ra, − 0.997 for ^232^Th, and + 1.057 for ^40^K indicate that the activity concentration distributions deviate from perfect normality and exhibit different degrees of asymmetry. The positive skewness observed for ²²⁶Ra and ⁴⁰K indicates that their distributions are right-skewed, meaning that most samples have relatively low to moderate activity concentrations, with a few samples showing comparatively higher values. Whereas, the negative skewness of ^232^Th indicates a left-skewed distribution, indicating a limited number of samples with lower activity values relative to the mean. The Kurtosis values for ^226^Ra, ^232^Th and ^40^K + 0.397, + 0.632 and +0.156, all approximating zero, indicating that the distributions are nearly normal.

Table 2 demonstrates comparison of present work with previously done similar works of some countries all around the world.

**Table 2:** Comparison of present work with previous work around the world.

Country/Region	^226^Ra (Bqkg^-1^)	^232^Th (Bqkg^-1^)	^40^K (Bqkg^-1^)	Type of sample	Detector	References
Japan	18.1	25.9	535.6	Urban and rural soil	HPGe	[39]
Turkey	60.1	50.1	631	Urban soil	HPGe	[40]
Saudi Arabia	14.5	11.2	225	Rural soil	HPGe	[41]
India	32.4	37.21	251.61	Rural soil	NaI (Tl)	[42]
China	66	109	211	Semi-rural soil	HPGe	[43]
Ghana	20.9	43.8	140.6	Urban soil	HPGe	[44]
Nigeria	64.6	72.0	680.6	Rural soil	HPGe	[45]
Mexico	42	51	811	Urban soil	NaI (Tl)	[46]
Malaysia	82	123	643	Rural soil	HPGe	[47]
Pakistan	37	43.0	737	Urban soil	HPGe	[48]
Tunisia	27	13	264	Rural-industrial soil	HPGe	[49]
Cameroon	23.8	72	105	Rural soil	NaI (Tl)	[50]
Dhaka, Bangladesh	24.2	52.0	352.3	Urban soil	HPGe	Present work
World average (UNSCARE, 2000)	35	30	400	–	–	[6]

Table 2 presents a comparison of the activity concentrations of ^226^Ra, ^232^Th, and ^40^K in soil from different countries worldwide, together with the results of the present study conducted in Dhaka, Bangladesh. To ensure consistency and avoid selective bias, only one representative dataset per country is included, and all values refer to surface soil measured using gamma-ray spectrometry. The activity concentrations of ^226^Ra and ^40^K observed in Dhaka soil are similar to the reported values of Japan, Saudi Arabia, Ghana, and Tunisia, while lower than values reported from China, Nigeria, Malaysia, and Turkey; however, they are comparable to Turkey and Mexico. The ^232^Th concentration in Dhaka soil is slightly higher than in Japan, Saudi Arabia, India, Tunisia and the UNSCEAR world average value but lower than in China, Nigeria, Malaysia and Cameroon. The variations of radionuclides may be due to the geological circumstances, topographical characteristics, rock formation, mineralogical features, and soil conditions, weathering can all contribute to changes in radionuclides [5,14]. A number of variables, such as industrial processes, building materials, human activity, and the redistribution of naturally existing radionuclides, instructional waste practice, presence of thorium rich mineral may be responsible for the higher ^232^Th concentrations found in Dhaka soil [18,23,51]. The physical, chemical, and geological structure and location features of soil samples vary with human activities, making the results very location and area specific [29,52,53].

### 3.2 Radiological hazard indices

Table 3 demonstrates radium equivalent activity (Ra_**eq**_), outdoor absorbed dose, indoor absorbed dose, outdoor annual effective dose and indoor annual effective dose of all the soil samples.

**Table 3:** Radium equivalent activity, annual effective dose rate, excess lifetime cancer risk and hazard indices of soil samples.

Sample ID	Ra_eq_ (Bqkg^-1^)	Outdoor absorbed dose (nGyhr^-1^)	Outdoor annual effective dose (mSvy^-1^)	ELCR $\times {\text{1}\text{0}}^{-\text{0}\text{4}}$
N1	121.55	55.47	0.068	2.38
N2	132.40	61.39	0.075	2.64
N3	120.06	54.35	0.067	2.33
N4	118.92	53.71	0.066	2.31
N5	143.36	65.76	0.081	2.82
N6	125.45	56.68	0.070	2.43
N7	124.37	57.73	0.071	2.48
N8	154.46	71.49	0.088	3.07
N9	129.03	58.34	0.072	2.50
N10	123.71	55.83	0.068	2.40
N11	136.22	62.34	0.076	2.68
N12	125.86	57.05	0.070	2.45
N13	146.38	66.32	0.081	2.85
N14	94.83	43.91	0.054	1.88
N15	137.15	62.09	0.076	2.67
N16	84.05	38.96	0.048	1.67
N17	141.69	64.31	0.079	2.76
N18	127.26	58.30	0.072	2.50
N19	151.81	68.77	0.084	2.95
N20	94.13	43.27	0.053	1.86
N21	107.96	48.75	0.060	2.09
N22	96.74	44.97	0.055	1.93
N23	136.78	61.87	0.076	2.66
N24	126.38	57.13	0.070	2.45
N25	134.67	60.72	0.074	2.61
N26	121.55	54.87	0.067	2.36
N27	148.33	66.93	0.082	2.87
N28	126.25	57.13	0.070	2.45
N29	127.68	59.45	0.073	2.55
N30	113.12	51.09	0.063	2.19
Average	125.74	57.30	0.070	2.46

From Table 3, it has been observed that radium equivalent activity of investigated urban soil samples ranged from 84.05 to 154.46 Bqkg^-1^ with the mean value of 125.74 Bqkg^-1^ which is less than the world average value of 370 Bqkg^-1^ [6]. The range of outdoor absorbed dose rates was found to be 38.96 to 71.49 nGyhr^-1^. The average value (57.30 nGyhr^-1^) of outdoor absorbed dose rates was found lower than the world average values of 59 nGyhr^-1^ [6]. The outdoor annual effective dose rate can be found in the range of 0.048 to 0.088 mSvy^-1^ with an average value of 0.070 mSvy^-1^, and in comparison, with the world average value, it is within the world recommended limit which indicates there is no significant radiological risks to the local community or ecosystem from the soil. The range of ELCR vale was found 1.67$\times {\text{1}\text{0}}^{-\text{0}\text{4}}$ to 3.07$\times {\text{1}\text{0}}^{-\text{0}\text{4}}.\;$The average value of excess lifetime cancer risk (ELCR) was 2.46$\times {\text{1}\text{0}}^{-\text{0}\text{4}}\;$and below than the world recommended value of 0.29$\times {\text{1}\text{0}}^{-\text{3}}\;$indicating there is no matter of concern toward the overall public health but monitoring is important [6,54].

The descriptive statistics of ^226^Ra, ^232^Th and ^40^K and radium equivalent activity, absorbed dose, annual effective dose and ELCR for urban soil of Dhaka city presented in Table 4.

**Table 4:** Mean, median, SD, minimum and maximum of ^226^Ra, ^232^Th and ^40^K and radium equivalent activity, absorbed dose, annual effective dose and ELCR for urban soil.

Parameter	^226^Ra (Bqkg^-1^)	^232^Th (Bqkg^-1^)	^40^K (Bqkg^-1^)	${𝐑𝐚}_{eq}$ (Bqkg^-1^)	${𝐃}_{out}$ (nGyhr^-1^)	${𝐀𝐄𝐃𝐄}_{Out}$ (mSvy^-1^)	ELCR
Minimum	13.8	32.0	253.0	84.0	38.9	0.048	$\text{1}.\text{67}\times {\text{10}}^{-\text{4}}$
Maximum	35.0	66.0	530.0	154.4	71.4	0.088	$\text{3}.\text{07}\times {\text{10}}^{-\text{4}}$
Mean	24.2	52.0	352.3	125.7	57.3	0.070	$\text{2}.\text{46}\times {\text{10}}^{-\text{4}}$
Median	23.7	54.5	325.0	126.3	57.4	0.070	$\text{2}.\text{47}\times {\text{10}}^{-\text{4}}$
SD	4.8	8.3	74.0	16.9	7.62	0.009	$\text{3}.\text{27}\times {\text{10}}^{-\text{5}}$

Table 4 shows the minimum, maximum, median, mean, SD, skewness and Kurtosis of ^226^Ra, ^232^Th and ^40^K and Ra_eq_, absorbed dose, annual effective dose and ELCR for urban soil of Dhaka city. From the minimum and maximum values, it is seen that the radionuclide concentration exhibits a wide range of variations across the urban soil samples. The median is near the mean for all radionuclides, and the mean value is higher than the standard deviation, suggesting less variability.

### 3.3 Analysis of frequency distributions and Q-Q plots

**Fig 2:**
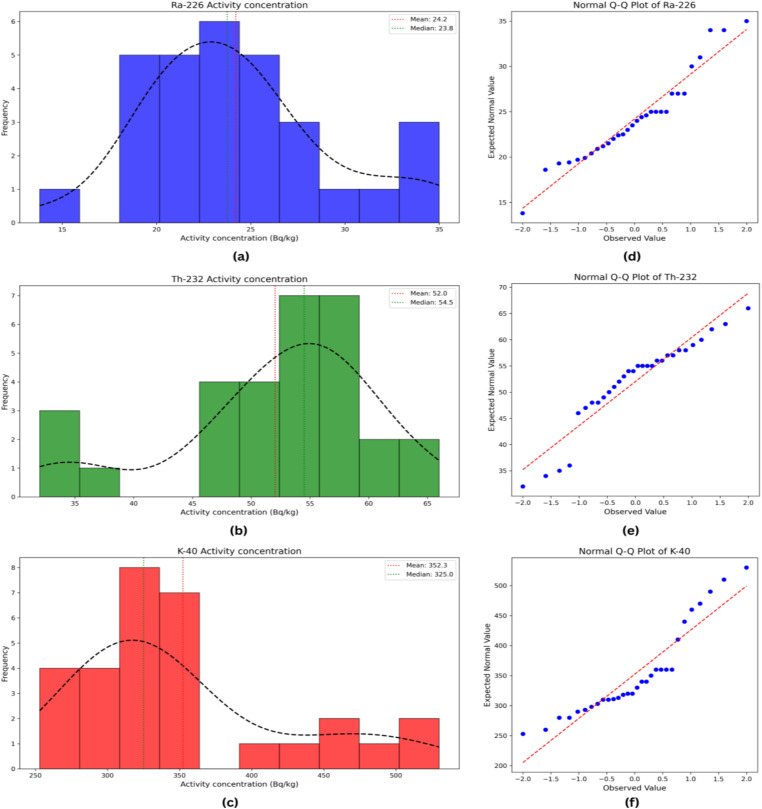
Frequency distribution (Fig 2a–c) and normal Q-Q plots (Fig 2d–f) analysis of ^226^Ra, ^232^Th and ^40^K in urban soil samples from Dhaka city.

The distributions of radionuclide activity concentrations in urban soil samples from Dhaka city were assessed using histograms and normal Q–Q plots generated with Python 3.13.3, employing its mathematical and visualization libraries (Fig 2). The histograms (Fig 2a–2c) indicate that the activity concentrations of ²³²Th, and ⁴⁰K were asymmetric, showing varying degrees of skewness and multimodality. Specifically, ²²⁶Ra and ⁴⁰K exhibited positive skewness, while ²³²Th showed slight negative skewness. These multimodal patterns likely reflect the heterogeneous mineralogical composition of the soils, which may influence gamma radiation emission and potentially affect localized radiological exposure.

The normal Q–Q plots (Fig 2d–2f) corroborated these observations, as data points for all three radionuclides deviated from the 45° reference line, particularly in the tails, indicating deviations from normality. Despite these deviations, no pronounced extreme outliers were observed for ²²⁶Ra, ²³²Th, and ⁴⁰K, suggesting the absence of anomalous contamination within the study area.

Overall, the combined histogram and Q–Q plot analyses indicate that although the radionuclide distributions are not strictly normal, the activity concentrations show consistent patterns without extreme outliers. The observed skewness and multimodality likely arise from soil heterogeneity, which is a key factor in understanding the environmental distribution and radiological characteristics of ²²⁶Ra, ²³²Th, and ⁴⁰K in the study area. These findings align with previous studies [55,56], highlighting the importance of site-specific assessment in radiological risk evaluation.

The Shapiro–Wilk test for ²²⁶Ra, ²³²Th, and ⁴⁰K was performed in Table 1 to support the clarifications of frequency distributions and Q–Q plots of the obtained values. As it has been found that all *p*-values were greater than 0.05, the data did not significantly deviate from a normal distribution. This formally supports the findings from the Q–Q plots and frequency distributions.

## 4. Conclusion

Thirty urban soil samples from Bangladesh’s most polluted city were used in this study to determine the activity concentrations of ^226^Ra, ^232^Th, and ^40^K as well as the related radiological health risk. The results showed that ^232^Th and ^40^K are the primary determinants affecting radiological exposure and risk in urban soils, with ^226^Ra having a negligible effect. The variations of radionuclides activity concentration may be the industrial activities and characteristics of the soil environment of Dhaka city. While the activity levels of ^226^Ra and ^40^K fall within the internationally recommended safety limits, the concentration of ^232^Th exceeds limit. However, However, the radium equivalent activity remained below the safe limit. Also, the study calculated an annual effective dose rate (0.070 mSvy^-1^) was found below the UNSCEAR limits of 1mSvy^-1^. The excess lifetime cancer risk (ELCR) was also within the permissible global threshold. Notably, no artificial radionuclide such as ^137^Cs was detected, suggesting the absence of nuclear contamination or anthropogenic radioactive sources near the study area. The findings of this study suggest continuous monitoring of soil radioactivity in Greater Dhaka to ensure the radiological safety of the residents of this city. Additionally, this study presents the first systematic baseline data, incorporating radiological health risk assessment and statistical analysis, to inform policy making and future radiological mapping and risk management as Bangladesh prepares to operate the Rooppur Nuclear Power Plant.

## References

[1] El Samad O, Baydoun R, Nsouli B, Darwish T. Determination of natural and artificial radioactivity in soil at North Lebanon province. J Environ Radioact. 2013;125:36–9. doi: 10.1016/j.jenvrad.2013.02.010 23498968

[2] Günay O, Eke C. Determination of terrestrial radiation level and radiological parameters of soil samples from Sariyer-Istanbul in Turkey. Arab J Geosci. 2019;12(20):631. doi: 10.1007/s12517-019-4830-1

[3] Khandaker MU, Heffny N ’Adillah B, Amin YM, Bradley DA. Elevated concentration of radioactive potassium in edible algae cultivated in Malaysian seas and estimation of ingestion dose to humans. Algal Research. 2019;38:101386. doi: 10.1016/j.algal.2018.101386

[4] Wong E, Tan HJ, Corcho-Alvarado JA, Loh E, Ong J, Ong CY, et al. Natural and anthropogenic radionuclides in selected environmental radioactivity monitoring sites in Singapore. J Radioanal Nucl Chem. 2025;334(2):1433–43. doi: 10.1007/s10967-024-09920-w

[5] Agbalagba EO, Avwiri GO, Chad-Umoreh YE. γ-Spectroscopy measurement of natural radioactivity and assessment of radiation hazard indices in soil samples from oil fields environment of Delta State, Nigeria. J Environ Radioact. 2012;109:64–70. doi: 10.1016/j.jenvrad.2011.10.012 22310017

[6] UNSCEAR. Sources and Effects of Ionizing Radiation. New York, USA: United Nations; 2000.

[7] Qadr HM. Radiological hazard assessment due to natural radioactivity content in cement material used in Iraqi Kurdistan region. Acta Geophys. 2024;73(1):549–56. doi: 10.1007/s11600-024-01480-7

[8] Kanmi AS, Ibrahim U, Goki NG, Rilwan U, Sayyed MI, Wais TY, et al. Estimation of soil-to-plant transfer factor across six local government areas of Kwara state, Nigeria. J Environ Radioact. 2024;280:107548. doi: 10.1016/j.jenvrad.2024.107548 39362113

[9] Fathy D, Zakaly HMH, Lasheen ESR, Elsaman R, Alarifi SS, Sami M, et al. Assessing geochemical and natural radioactivity impacts of Hamadat phosphatic mine through radiological indices. PLoS One. 2023;18(8):e0287422. doi: 10.1371/journal.pone.0287422 37535632 PMC10399901

[10] Lasheen ESR, Saleh GM, Al-Mur BA, Abdelaal A. Assessing the radioactive properties and environmental risks of Hankorab sediments on the Red Sea coast. Environ Earth Sci. 2025;84(14). doi: 10.1007/s12665-025-12418-7

[11] Eke BC, Akomolafe IR, Ukewuihe UM, Onyenegecha CP. Assessment of radiation hazard indices due to natural radionuclides in soil samples from Imo State University, Owerri, Nigeria. Environ Health Insights. 2024;18:1–11. doi: 10.1177/11786302231224581 PMC10826396 38292566

[12] Eke BC, Ukewuihe UM, Akomolafe IR. Evaluation of activity concentration of natural radionuclides and lifetime cancer risk in soil samples at two tertiary institutions in Owerri, Imo State, Nigeria. Int J Radiat Res. 2022;20(3):671–8. doi: 10.52547/ijrr.20.3.22

[13] Pourimani R, Shahroodi SMM. Radiological assessment of the artificial and natural radionuclide concentrations of wheat and barley samples in Karbala, Iraq. Iran J Med Phys. 2018;15(2):126–31. doi: 10.22038/ijmp.2017.24190.1238

[14] Bharath KM, Bramha S, Chandrasekaran S, Krishnaveni M. Geospatial assessment of natural radionuclides in urban soil of Chennai Metropolitan City. Sci Rep. 2025;15(1):40899. doi: 10.1038/s41598-025-24681-6 41258355 PMC12630799

[15] Taqi AH, Shaker AM, Battawy AA. Natural radioactivity assessment in soil samples from Kirkuk city of Iraq using HPGe detector. Int J Radiat Res. 2018;16(4):455–63. doi: 10.18869/acadpub.ijrr.16.4.455

[16] Rahman S, Faheem M. Natural radioactivity measurements in Pakistan--an overview. J Radiol Prot. 2008;28(4):443–52. doi: 10.1088/0952-4746/28/4/R01 19029595

[17] Jibiri NN, Ugbechie A, Sowunmi AA, Akomolafe IR. Radionuclide contents in sediment and seafood from Makoko Lagoon, Lagos State, Nigeria. Mar Pollut Bull. 2023;192:1–12. 10.1016/j.marpolbul.2023.114992 37182242

[18] Kanmi AS, Ibrahim U, Goki NG, Rilwan U, Sayyed MI, Maghrbi Y. Assessment of natural radioactivity and its radiological risks in the soil of local government areas (Asa, Ilorin East, Ilorin South, Irepodun, Moro, and Oyun) in Kwara State, Nigeria. Case Studies in Chemical and Environmental Engineering. 2025;11:1–12. doi: 10.1016/j.cscee.2024.101040

[19] Singh S, Singh B, Kumar A. Natural radioactivity measurements in soil samples from Hamirpur district, Himachal Pradesh, India. Radiat Meas. 2003;36(1–6):547–9. doi: 10.1016/S1350-4487(03)00200-2

[20] Zakaly HMH, Uosif MAM, Issa SAM, Tekin HO, Madkour H, Tammam M, et al. An extended assessment of natural radioactivity in the sediments of the mid-region of the Egyptian Red Sea coast. Mar Pollut Bull. 2021;171:112658. doi: 10.1016/j.marpolbul.2021.112658 34271507

[21] Eke BC, Amakom CM, Ukewuihe UM, Akomolafe IR, Ejelonu BO, Okereke BO. Radiological dose assessment due to the presence of norms in the top soil of the imo state polytechnic, imo State Nigeria. Environ Health Insights. 2022;16:11786302221137219. doi: 10.1177/11786302221137219 36408334 PMC9666849

[22] Hannan M, Wahid K, Nguyen N. Assessment of natural and artificial radionuclides in Mission (Texas) surface soils. J Radioanal Nucl Chem. 2015;305(2):573–82. doi: 10.1007/s10967-015-4018-4

[23] Demewoz NM, Kassie LN, Zeleke HG. Assessment of radioactivity in soil samples from Wolaita Sodo town, Ethiopia: implications for environmental and public health. Radiat Prot Dosimetry. 2025;201(3):160–77. doi: 10.1093/rpd/ncaf002 39848233 PMC11884513

[24] Abedin MJ, Khan R. Primordial radionuclides in the dust samples from the educational institutions of central Bangladesh: radiological risk assessment. Heliyon. 2022;8(11):e11446. doi: 10.1016/j.heliyon.2022.e11446 36387448 PMC9647501

[25] Barnekow U, Fesenko S, Kashparov V, Kis-Benedek G, Matisoff G, Onda YU. Guidelines on soil and vegetation sampling for radiological monitoring. Vienna: International Atomic Energy Agency (IAEA); 2019.

[26] International Atomic Energy Agency (IAEA). A guidebook. Measurement of radionuclides in food and the environment. Vienna: International Atomic Energy Agency; 1989.

[27] Yeasmin S, Das SK, Siraz MMM, Rahman AFMM, Rahman MS. Radiometric hazard assessment of soil and water samples adjacent to Bangladesh’s first nuclear power plant before commissioning: Insights into human health and environmental radiological dynamics. Heliyon. 2024;10(20):e39516. doi: 10.1016/j.heliyon.2024.e39516 39469689 PMC11513540

[28] Yang J, Sun Y. Natural radioactivity and dose assessment in surface soil from Guangdong, a high background radiation province in China. J Radiat Res Appl Sci. 2022;15(1):145–51. doi: 10.1016/j.jrras.2022.01.019

[29] Hossain S, Pervin S, Lubna L, Karmaker S, Yeasmin S, Khandaker MU. Transfer factors of naturally occurring radionuclides from soil-to-rice cultivated in Bangladesh and associated health implications. Heliyon. 2024;10(19):e38004. doi: 10.1016/j.heliyon.2024.e38004 39386782 PMC11462239

[30] Khandaker MU, Uwatse OB, Bin Shamsul Khairi KA, Faruque MRI, Bradley DA. Terrestrial radionuclides in surface (DAM) water and concomitant dose in metropolitan kuala lumpur. Radiat Prot Dosimetry. 2019;185(3):343–50. doi: 10.1093/rpd/ncz018 30806465

[31] Kanmi AS, Wais TY, Namq BF, Najam LA, Ibrahim U, Tanko IY, et al. Assessment of natural radioactivity and radiological risks associated with African spinach from Kwara State, Nigeria. Eur Phys J Plus. 2025;140(7). doi: 10.1140/epjp/s13360-025-06560-1

[32] Duong V-H, Santhanabharathi B, Pradhoshini KP, Trung TC, Ngo TTH, Nguyen T-M, et al. Radiological hazard assessments of natural radionuclides in surface soil in Chau Binh, Vietnam. Environmental Pollution and Management. 2025;2:172–81. doi: 10.1016/j.epm.2025.07.002

[33] Adedokun MB, Aweda MA, Maleka PP, Obed RI, Ibitoye AZ. Evaluation of natural radionuclides and associated radiation hazard indices in soil and water from selected vegetable farmlands in Lagos, Nigeria. Environmental Forensics. 2020;23(3–4):301–13. doi: 10.1080/15275922.2020.1850557

[34] Younis H, Shafique S, Ehsan Z, Ishfaq A, Mehboob K, Ajaz M. Radiometric examination of fertilizers and assessment of their health hazards, commonly used in Pakistan. Nucl Eng and Technol. 2023;55(7):2447–53. doi: 10.1016/j.net.2023.03.020

[35] Bangou C, Otoo F, Darko EO. Performance testing and comparative study of natural radioactivity in soil samples using high purity germanium (HPGe) detector. MethodsX. 2021;8:101397. doi: 10.1016/j.mex.2021.101397 34430293 PMC8374471

[36] Ibikunle SB, Arogunjo AM, Ajayi OS. Characterization of radiation dose and soil-to-plant transfer factor of natural radionuclides in some cities from south-western Nigeria and its effect on man. Sci Afr. 2019;3(May):1–10. doi: 10.1016/j.sciaf.2019.e00062

[37] ICRP. Recommendations of the International Commission on Radiological Protection. 60. Oxford, UK: Pergamon Press; 1990.

[38] Demewoz NM, Kassie LN, Zeleke HG. Assessment of radioactivity in soil samples from Wolaita Sodo town, Ethiopia: implications for environmental and public health. Radiat Prot Dosimetry. 2025;201(3):160–77. doi: 10.1093/rpd/ncaf002 39848233 PMC11884513

[39] Sahoo SK, Hosoda M, Kamagata S, Sorimachi A, Ishikawa T, Tokonami S, et al. Thorium, Uranium and Rare Earth Elements Concentration in Weathered Japanese Soil Samples. Progress in Nuclear Science and Technology. 2011;1(0):416–9. doi: 10.15669/pnst.1.416

[40] Turhan Ş. Radiological assessment of urban soil samples in the residents of a central Anatolian volcanic province, Turkey. Int J Environ Health Res. 2023;33(12):1181–94. doi: 10.1080/09603123.2022.2078797 35594037

[41] Alotaibi MF, Alharbi KN, Alosime EM, Alhawali LH, Albarqi MM, Alsulami RA. Natural radioactivity in soil and water of Saudi Arabia: A mixed-studies review. J Radiat Res Appl Sci. 2024;17(2):1–19. doi: 10.1016/j.jrras.2024.100897

[42] Mugalgaon RS, Mugalgaon AR, Kerur BR. Measurement of natural radioactivity levels in soil samples of Bidar district, Karnataka, India. Radiat Prot Dosimetry. 2024;200(11–12):1059–63. doi: 10.1093/rpd/ncae025 39016509

[43] Yang B, Pang C, Tuo F, Zhou Q, Li Z. Radioactivity and elemental oxidation composition in soil from Yangjiang, a high background natural radiation area, China. J Environ Radioact. 2024;276:107447. doi: 10.1016/j.jenvrad.2024.107447 38749216

[44] Annan RAT, Eghan MJ, Amoako JK, Otoo F, Adotey DK, Opoku-Ntim I, et al. Radiological impact assessment of natural radioactivity in soil and water in Cape Coast North, Central Region of Ghana. Radiat Prot Dosimetry. 2024;200(15):1450–61. doi: 10.1093/rpd/ncae188 39242111

[45] Muhammad AN, Ismail AF, Garba NN. Natural radioactivity in food crops and soil and estimation of the concomitant dose from tin mining areas in Nigeria. Journal of Taibah University for Science. 2024;18(1). doi: 10.1080/16583655.2024.2366507

[46] Mandujano-García CD, Sosa M, Mantero J, Costilla R, Manjón G, García-Tenorio R. Radiological impact of natural radionuclides from soils of Salamanca, Mexico. Appl Radiat Isot. 2016;117:91–5. doi: 10.1016/j.apradiso.2016.01.031 26867693

[47] Garba NN, Ramli AT, Saleh MA, Gabdo HT. Natural radioactivity and associated radiation hazards in soil of Kelantan, Malaysia. Human and Ecological Risk Assessment: An International Journal. 2018;25(7):1707–17. doi: 10.1080/10807039.2018.1474433

[48] Ghias S, Satti KH, Khan M, Dilband M, Naseem A, Jabbar A, et al. Health risk assessment of radioactive footprints of the urban soils in the residents of Dera Ghazi Khan, Pakistan. Chemosphere. 2021;267:129171. doi: 10.1016/j.chemosphere.2020.129171 33348265

[49] Machraoui S, Labidi S, Purushotham MM. Assessment of gamma absorbed doses and radiological risk indexes from soil radioactivity around the phosphate area in south Tunisia. Radiat Prot Dosimetry. 2024;200(4):387–95. doi: 10.1093/rpd/ncad299 38186062

[50] Tiomene DF, Bongue D, Ngwa Ebongue A, Haman F, Penabei S, Guembou Shouop CJ, et al. Radionuclides distribution in soils and radon level assessment in dwellings of Mungo and Nkam Divisions, Cameroon. Environ Monit Assess. 2024;196(11):1038. doi: 10.1007/s10661-024-13158-0 39384632

[51] Siraz MMM, Islam S, Shelley A, Alam MS, Mahmud A, Rashid MB, et al. Radioactivity distribution and concomitant hazards evaluation of industrial zones soils from Chattogram, Bangladesh: A multivariate statistical analysis. PLoS One. 2025;20(7):e0328356. doi: 10.1371/journal.pone.0328356 40668792 PMC12266464

[52] Aközcan Pehlivanoğlu S, Mancini S, Özden S, Guida M, Falanga M. Characterization of a typical urban soil in terms of natural radionuclide content. The case study of a university campus. Heliyon. 2024;10(17):e37145. doi: 10.1016/j.heliyon.2024.e37145 39296170 PMC11409136

[53] Chandra K, Proshad R, Dey HC, Idris AM. A review on radionuclide pollution in global soils with environmental and health hazards evaluation. Environ Geochem Health. 2023;45(12):9245–66. doi: 10.1007/s10653-023-01725-2 37578560

[54] Dizman S, Görür FK, Keser R. Determination of radioactivity levels of soil samples and the excess of lifetime cancer risk in Rize province, Turkey. Int J Radiat Res. 2016;14(3):237–44. doi: 10.18869/acadpub.ijrr.14.3.237

[55] Mowafaq Marie Z., Najam L, Yaseen Wais T., Namq F. Radionuclides distribution in Al-Qayyarah oil wells and refinery in nineveh government, Iraq. International Journal of Engineering and Applied Physics. 2024;4(3):1055–67.

[56] Ashraf M, Radha CA, Ahmad S, Masood S, Dar RA, Ramasubramanian V, et al. Radiological health assessment due to gamma radiation levels of natural radioactivity of soil in vicinity of Nichahoma lignite belt, Kashmir Valley. Radiochimica Acta. 2016;104(6):435–44. doi: 10.1515/ract-2015-2498

